# A Ruptured Ectopic Pregnancy Presenting with a Negative Urine Pregnancy Test

**DOI:** 10.1155/2016/7154713

**Published:** 2016-09-07

**Authors:** Johnathan Michael Sheele, Rachel Bernstein, Francis L. Counselman

**Affiliations:** ^1^Department of Emergency Medicine, University Hospitals Case Medical Center, Cleveland, OH 44106, USA; ^2^New York-Presbyterian Hospital/Columbia University Medical Center, New York, NY 10032, USA; ^3^Department of Emergency Medicine, Eastern Virginia Medical School and Emergency Physicians of Tidewater, Norfolk, VA 23507, USA

## Abstract

A negative urine pregnancy test in the emergency department traditionally excludes the diagnosis of pregnancy. We report a rare case of ruptured ectopic pregnancy in a patient with a negative urine pregnancy test but with a serum beta-human chorionic gonadotropin (*β*-hCG) of 10 mIU/mL. The patient developed hemoperitoneum and required laparoscopy by Obstetrics and Gynecology (OB/Gyn). This case highlights the fallibility of the urine pregnancy test in diagnosing early pregnancy.

## 1. Introduction

Ectopic pregnancy remains a leading cause of death in women of childbearing age in the United States [1, 2]. Women at highest risk include those less than 25 years of age and of nonwhite ethnicity [1, 2]. In the emergency department (ED), the prompt identification of a pregnant woman with an ectopic pregnancy is critical because the sudden rupture of a fallopian tube can lead to hemorrhagic shock [1, 2]. In addition, early diagnosis may allow for nonoperative intervention and preservation of fertility. The classic triad for an ectopic pregnancy of abdominal pain, amenorrhea, and vaginal bleeding is only present in about 50% of women with this condition.

## 2. Case Presentation

A 35-year-old woman with a past medical history of bipolar affective disorder, anxiety, hemorrhoids, and polysubstance abuse presented to the ED with the chief complaint of rectal discomfort. She had two days of diffuse abdominal pain radiating to the lower back, dyspareunia, dyschezia, and nausea without vomiting. She denied urinary complaints, vaginal discharge, or bleeding. Her last menstrual period was four weeks before.

Her physical exam—including pelvic, rectal, and abdominal exam—was unremarkable, and her vital signs were stable. Urinalysis, complete blood count (CBC), basic metabolic panel (BMP), and a vaginal wet prep were all within normal limits. Her urine pregnancy test was “weakly positive” and her serum *β*-hCG was 23 mIU/mL. A pelvic ultrasound showed no evidence of a gestational sac and was otherwise unremarkable. She was diagnosed with early pregnancy and constipation and referred for serial *β*-hCG measurements and repeat pelvic imaging.

**Figure 1:**
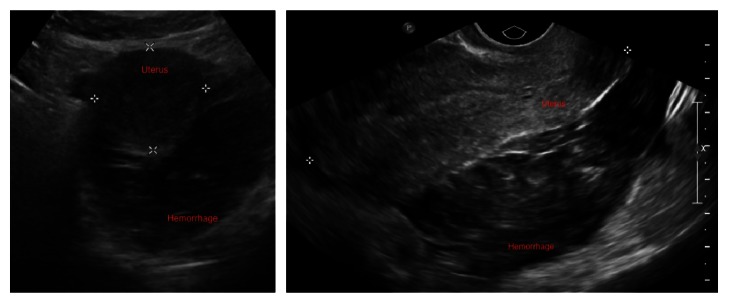
Transverse and longitudinal ultrasound images of the uterus showing intra-abdominal hemorrhage and no intrauterine pregnancy.

She returned to our ED three days later with complaints of worsening abdominal pain, increasing nausea, and dysuria. She denied vaginal bleeding. Physical exam revealed stable vitals, severe diffuse abdominal pain with guarding, cervical motion tenderness, and bilateral adnexal tenderness without palpable masses. Her urine pregnancy test was negative but her serum *β*-hCG was 10 mIU/mL. Her hemoglobin had dropped from 13.2 g/dL three days earlier to 10.8 g/dL.  Figure 1 shows the repeat pelvic ultrasound images, demonstrating a large complex fluid collection in the pelvic cul-de-sac, possibly representing a hemorrhage without evidence of an intrauterine pregnancy. OB/Gyn emergently took the patient to the operating room for laparoscopy where she was diagnosed with hemoperitoneum and ruptured ectopic pregnancy. Gestational tissue was identified during the surgery. Her postoperative recovery was unremarkable.

## 3. Discussion

Diagnosing a ruptured ectopic pregnancy with a negative urine pregnancy test is exceptionally rare and only a few cases have been reported in the literature [3–8]. The following list summarizes reported cases of ectopic pregnancies with negative urine pregnancy tests.

Published reports involving ruptured ectopic pregnancy and a negative urine *β*-hCG test are as follows:

 Lee and Lamaro, 2009 [3]: 25-year-old with a *β*-hCG of 4 IU/L.
 Pabon et al., 2011 [4]: 34-year-old with a *β*-hCG of 6 IU/L.
 Nishijima et al., 2005 [5]: 32-year-old with a *β*-hCG of 1.84 IU/L.
 Kalinski and Guss, 2002 [6]: 44-year-old with a *β*-hCG of 7 IU/L.
 Brennan et al., 2000 [7]: 23- and 28-year-old, both with *β*-hCG levels of less than 25 IU/L.
 Grynberg et al., 2009 [8]: 26-year-old with both negative urine and serum *β*-hCG tests.
 Daniilidis et al., 2014 [9]: 36-year-old with a *β*-hCG of 13 IU/L.

Approximately 1% of ectopic pregnancies will have a negative urine pregnancy test and a *β*-hCG level of less than 20 mIU/mL. The emergency physician must remain cognizant of this potential diagnosis in the setting of unexplained intraabdominal hemorrhage or severe pelvic pain with a negative urine pregnancy test [2, 4].

In a normal intrauterine pregnancy, trophoblasts will secrete *β*-hCG with blood levels reaching 50–300 mIU/mL within two weeks of fertilization [10]. The urine pregnancy test will generally become positive when the serum *β*-hCG is greater than or equal to 25 mIU/mL [10]. In a normal early intrauterine pregnancy, the *β*-hCG level doubles approximately every 48–72 hours until about 60–90 days after conception [9]. Only 15% of women with ectopic pregnancies will have serum *β*-hCG levels that rise in a way similar to normal intrauterine pregnancies [9]. The most likely mechanism for low *β*-hCG levels in ectopic pregnancy is the degeneration of trophoblasts that result in cessation of *β*-hCG production [4]. Other causes can include a small number of chorionic villi present to produce *β*-hCG, abnormal *β*-hCG synthesis, or an enhanced *β*-hCG clearance [4]. A woman with an aborted pregnancy will have her *β*-hCG levels decreasing by approximately one-half in 48 hours and going to zero within several days [8].

Clinicians should not use the *β*-hCG level to determine the need for an ultrasound if a pregnant female has symptoms that may be consistent with an ectopic pregnancy. In one study, approximately 25% of pregnant women in the ED presenting with abdominal pain and/or vaginal bleeding were diagnosed with an ectopic pregnancy and a *β*-hCG less than 1500 mIU/mL, which has been the traditional *β*-hCG level at which an intrauterine pregnancy can be seen on ultrasound [11]. In a retrospective study of ectopic pregnancies, the authors found that 25% of patients had a *β*-hCG level less than 1000 mIU/mL, yet a pelvic ultrasound suspicious for ectopic pregnancy [12]. Our case illustrates the ongoing clinical diagnostic challenges associated with ectopic pregnancy. In the correct clinical setting, it is of importance not to exclude this potentially life-threatening diagnosis with a negative urine pregnancy test.
